# Abdominal wall defect repair with component separation technique for giant omphalocele with previous relaxing incisions on the abdominal skin

**DOI:** 10.1186/s40792-023-01679-8

**Published:** 2023-06-07

**Authors:** Makoto Matsukubo, Mitsuru Muto, Koji Yamada, Nanako Nishida, Chihiro Kedoin, Mayu Matsui, Ayaka Nagano, Masakazu Murakami, Koshiro Sugita, Keisuke Yano, Shun Onishi, Toshio Harumatsu, Waka Yamada, Takafumi Kawano, Tatsuru Kaji, Satoshi Ieiri

**Affiliations:** 1 Department of Pediatric Surgery, Kagoshima City Hospital, Kagoshima, Japan; 2 Department of Pediatric Surgery, Medical and Dental Area, Research and Education Assembly, Research Field in Medical and Health Sciences, Kagoshima University, 8-35-1, Sakuragaoka, Kagoshima City, 8908520 Japan; 3 Department of Pediatric Surgery, Kurume University School of Medicine, Kurume City, Fukuoka Japan

**Keywords:** Component separation technique, Giant omphalocele, Ventral hernia, Primary skin closure, Relaxing incisions

## Abstract

**Background:**

The repair of large abdominal wall defects that cannot be closed primarily is quite challenging. The component separation technique (CST) is a surgical approach using autologous tissue to close large abdominal wall defects. The CST requires extensive dissection between the abdominal skin and the anterior sheath of the rectus abdominis muscle. Subsequently, incisions are made at both sides of the external oblique aponeurosis, releasing the external oblique muscle from the internal oblique muscle, and then the right and left rectus abdominis muscles are brought together in the midline for defect closure. However, impairment of blood flow in the abdominal wall skin and necrotic changes are recognized as potential complications.

**Case presentation:**

The CST was performed in a 4-year-old boy with a large ventral hernia who had undergone skin closure with abdominal wall relaxing incisions for the primary treatment of giant omphalocele in the neonatal period. Given his history of incisions on the abdominal wall, he was speculated to be at high risk for postoperative skin ischemia. Dissection was therefore kept to a minimum to preserve the blood supply from the superior and inferior epigastric arteries and perforating branches of those arteries through the rectus abdominis muscle. In addition, care was taken to adjust the muscle relaxant dosage while monitoring the intravesical pressure, ensuring that it did not exceed 20 mmHg to avoid impaired circulation in the abdominal wall caused by abdominal compartment syndrome. He was discharged 23 days after the surgery without any complications, and neither recurrence of the ventral hernia nor bowel obstruction was observed in 4 years.

**Conclusions:**

A giant omphalocele with primary skin closure was treated by applying the CST. The procedure can be performed safely while preserving the blood flow to the abdominal wall, even in patients with a history of relaxing incisions on the abdominal skin. The CST is expected to be effective for repairing the large abdominal wall defects seen in giant omphalocele when primary closure is not possible.

## Background

The outcomes of congenital abdominal wall defects, including omphalocele, have remarkably improved thanks to advances in perinatal care and surgical techniques. However, the management of giant omphalocele with large defects and prolapse of the liver remains challenging [1, 2]. There is no one-size-fits-all treatment policy, and it must be flexibly adapted according to the patient’s condition.

We performed residual ventral hernia repair with the component separation technique (CST) [3] in a boy who had undergone direct skin closure with relaxing incisions on the abdominal wall for a giant omphalocele in the neonatal period, and obtained good results. The CST is considered to be an effective approach that expands the range of treatment options for giant omphaloceles.

## Case presentation

**Fig. 1:**
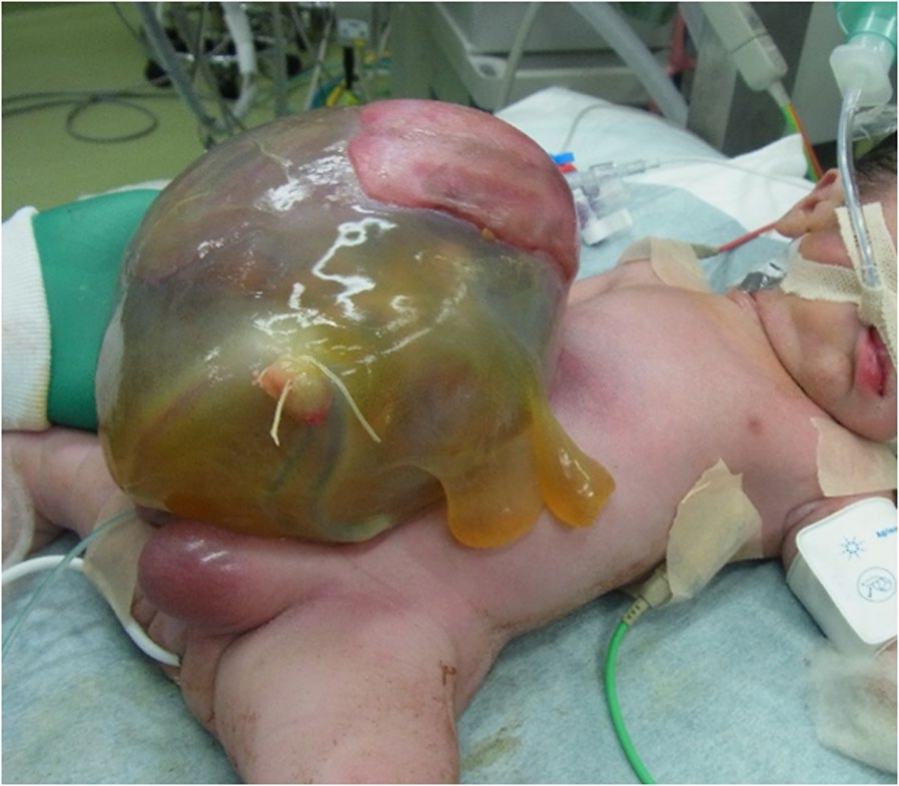
The appearance of omphalocele at birth. A giant omphalocele with whole-liver prolapse

**Fig. 2:**
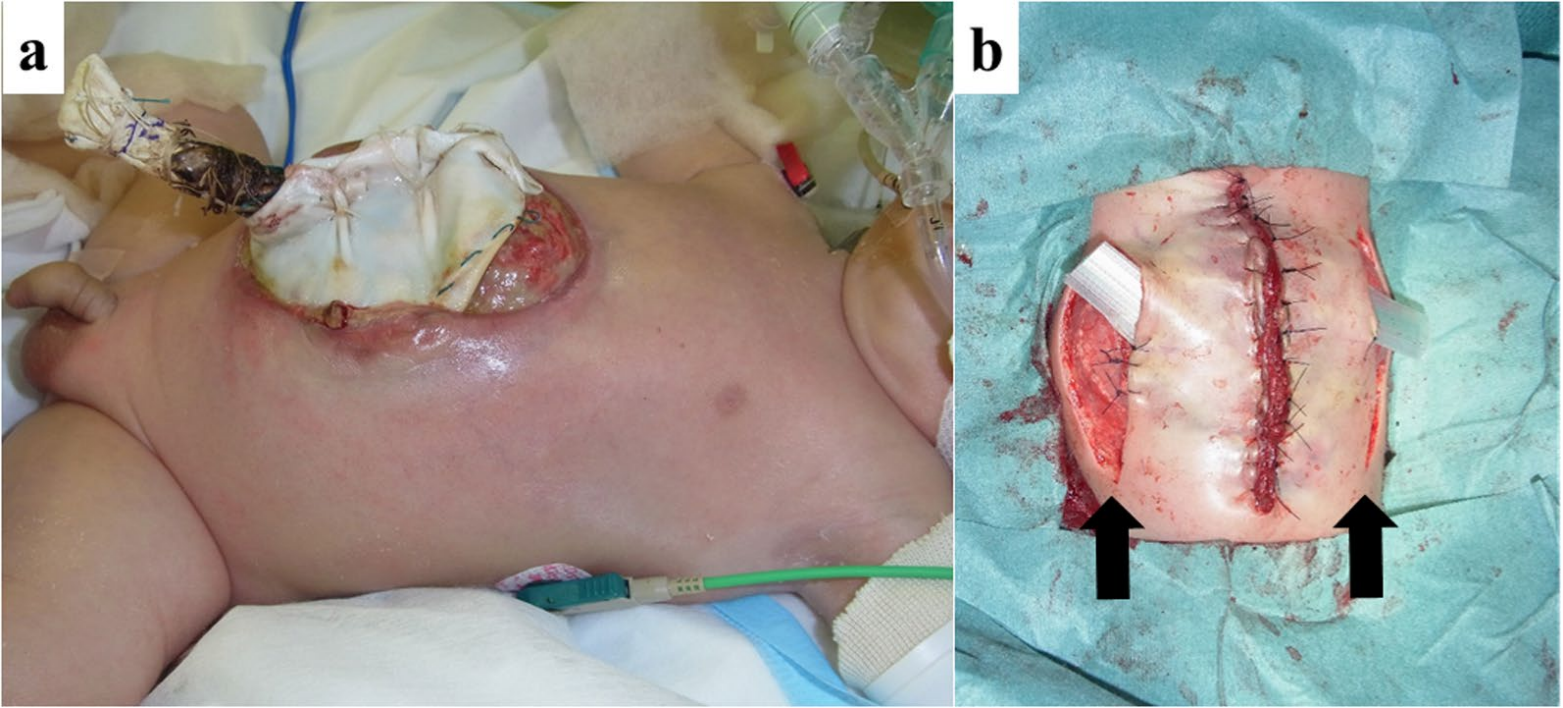
The appearance of the abdominal wall before and after emergent surgery in the neonatal period. **a** The oral side of the silo has detached and fallen off, becoming herniated, and the organs were exposed. **b** Two relaxing incisions (arrow) were made on the flanks, and primary skin closure was performed

The patient was a 4-year-old boy with prenatally diagnosed omphalocele. He had been born by emergency Cesarean section due to premature rupture of membranes at 37 weeks and 5 days of gestation, with a birth weight of 2500 g. A giant omphalocele (hernia orifice diameter 4 × 3 cm) with whole-liver prolapse was observed (Fig. 1). The hepatic vein was close to the hernia gate, and there was a concern that the ring of an XS size Alex Wound Retractor would compress the hepatic vein if it was used to create the silo. Then, silo construction using a Gore-Tex sheet according to the Allen-Wrenn method was performed on the day of birth. The oral side of the silo was detached during step-by-step reduction, and the herniated organs were exposed (Fig. 2a). It was difficult to close the muscle layers around the orifice, so direct closure of the skin with relaxing incisions was emergently performed (Fig. 2b). Impairment of the abdominal wall circulation was observed temporarily, and the patient was discharged at 73 days after birth.

**Fig. 3:**
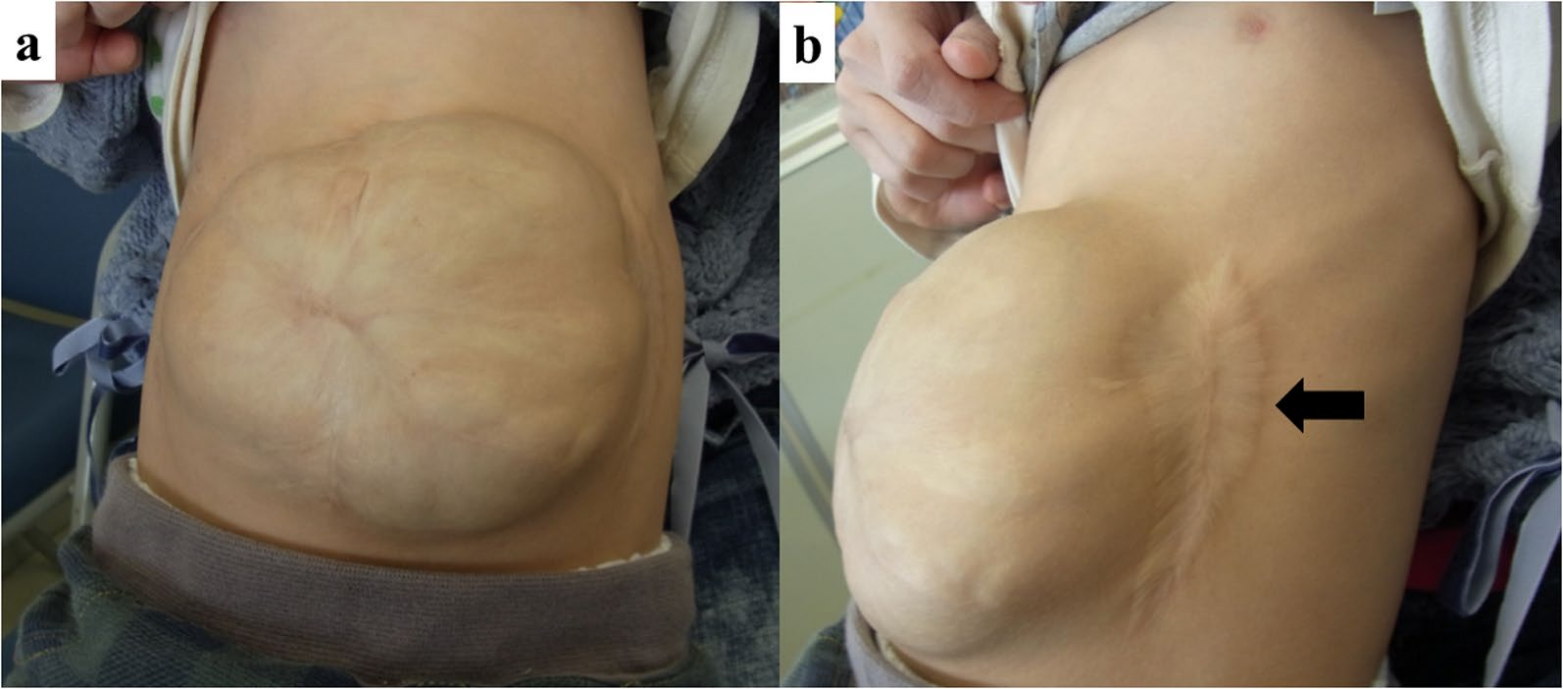
The appearance of the abdominal wall before repair with the CST at 4 years old. **a** Ventral herniation measuring 12 cm long and 10 cm wide was found centered on the umbilicus. **b** Surgical scars from the relaxing incision (arrow) were found on the flank

A huge ventral hernia remained (Fig. 3), and abdominal wall repair was attempted at 4 years of age when the patient weighed 12.1 kg. It was speculated that a history of relaxing incision may be a risk factor for the impairment of blood flow in the abdominal skin in abdominal wall repair using the CST. The policy was to preserve the feeding vessels of the abdominal wall skin as much as possible. Also, we afraid severe liver injury at the release of firm adhesion between the parietal peritoneum and the liver surface. Hence, the anterior CST was selected. A sufficient incision of the aponeurosis of the external oblique muscle with minimum lysis in the abdominal cavity allowed the large hernia to be more safely repaired. An 8Fr urethral catheter was inserted, and the intravesical pressure was monitored continuously with a pressure transducer during the perioperative period to detect elevated intra-abdominal pressure indirectly [4, 5]. Muscle relaxants were adjusted to control the abdominal wall tension for adequate blood flow preservation.

**Fig. 4:**
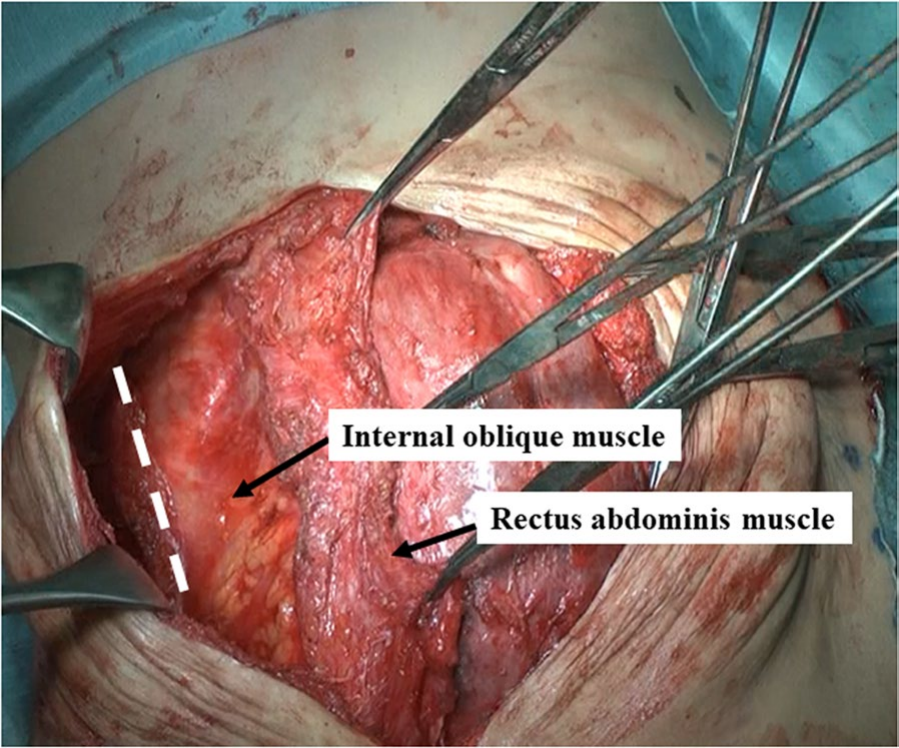
The appearance of the separated abdominal wall muscles. The rectus abdominis muscle was moved medially by adding a longitudinal incision to the aponeurosis of the external oblique muscle (dotted line)

A midline incision was made along the previous surgical wound from the xiphoid process to the lower abdomen. Lysis of adhesions between the liver surface and subcutaneous scar tissue was performed, taking care not to damage the liver. Subsequently, the outer edge of the external oblique muscle was exposed by dissecting the subcutaneous scar tissue and the anterior sheath of the rectus abdominis muscle. At this time, special care was taken to ensure preservation of the superior and inferior epigastric arteries and the perforator from the anterior sheath of the rectus abdominis muscle. The extent of the muscle layer defect was 12 × 10 cm. After exposing the lateral border of the rectus abdominis muscle, the area of its attachment to the aponeurosis of the external oblique muscle was dissected. Following relaxing incisions in the rectus sheath (Fig. 4) and pulling the right and left rectus muscles together at the midline, the intravesical pressure increased from 5 to 18 mmHg. Since it was below 20 mmHg, considered the acceptable upper limit [4], abdominal closure was completed. The intravesical bladder pressure increased to 20 mmHg on postoperative day (POD) 1, and the dose of muscle relaxants was increased. Thereafter the pressure decreased over time and was maintained at < 12 mmHg on POD 4. The administration of muscle relaxants was terminated, and extubation from the trachea was performed on POD 5. No complications were observed, and the patient was discharged from the hospital on POD 23.

**Fig. 5:**
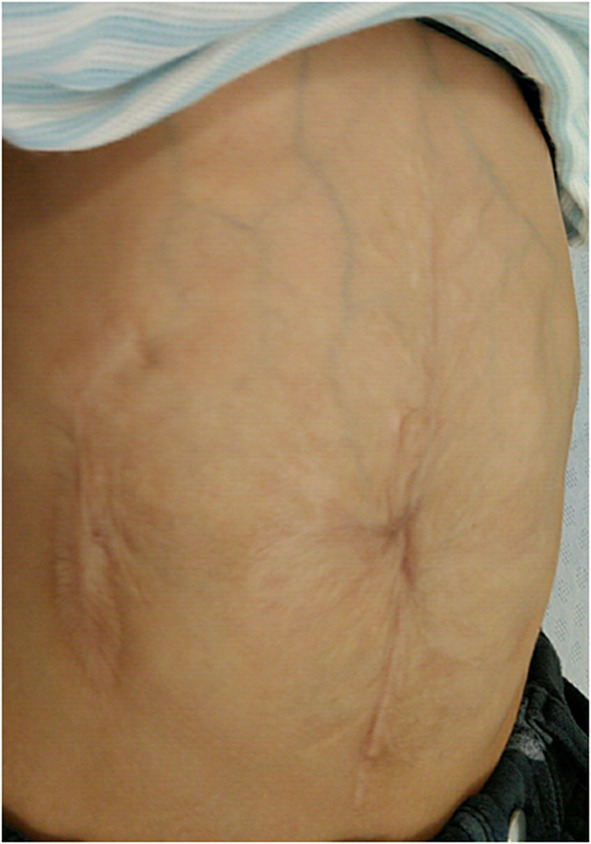
The appearance of the abdominal wall at 2 years after repair. The ventral hernia was completely healed, and no recurrence was observed

At 4 years after the surgery, neither ventral hernia recurrence nor adhesive bowel obstruction was observed (Fig. 5).

## Discussion

Repair of large abdominal wall defects that cannot be closed primarily, as in the present case of giant omphalocele with prolapsed whole liver, is quite challenging. Ramirez et al. first reported the CST in 1990 as a surgical approach using autologous tissue to close large abdominal wall defects [6]. They performed the CST in an adult case of giant ventral incisional hernia. In recent years, it has been widely applied to large open wounds on the abdominal wall resulting from trauma, severe burns, and similar issues [7–9].

There are two approaches to bring the rectus abdominis to the midline in the CST: “anterior” and “posterior” [2]. In the presented case, “anterior” CST was selected for the following reasons. In the “posterior” approach, the posterior rectus sheath is incised from the abdominal cavity, and the dorsal surface of the rectus abdominis muscle is dissected laterally toward the transversus abdominis muscle. Then, an incision is made in the transversus abdominis muscle, and dissection between the transversus abdominis muscle and transversalis fascia is maintained to free the rectus abdominis muscles to the midline, which seems superior to the “anterior” approach in terms of preserving the blood flow of the abdominal wall. However, there was concern about the risk of liver injury associated with broad lysis of firm adhesion between the liver and peritoneum required in the “posterior” approach and the difficulty of maintaining a good dissecting layer between the transversus abdominis muscle and transversalis fascia followed by lysis in the present case.

The first report of the application of the CST to children was reported by Wijnen et al. in 2005 [10], followed by subsequent case series reports [11–13]. Eijck et al. summarized the postoperative outcomes of 11 infants with giant omphalocele (defect diameter: 6–9 cm) who underwent CST at 5–69 months of age (median age: 6.5 months), and reported that the thickness and motor function of the abdominal wall muscle in their patients were not markedly different from those of normal children after 38–84 months (median: 54 months) [11, 14]. Although CST can presumably be safely performed after 6 months of age according to a review of the relevant literature, we believe that surgery should be performed once sufficient development of the abdominal wall muscles and growth of the intra-abdominal volume have been achieved in the patient.

Regarding postoperative complications associated with the CST, although findings are limited to adult cases, the following meta-analysis data have been reported: rate of wound infection, 18.9%; hematoma, 2.4%; seroma, 2.4%; abdominal wall skin necrosis, 1.5%; and recurrence of ventral hernia, 18.2% [15]. There was some concern about abdominal skin necrosis in the present case due to the history of relaxing incisions at the abdominal wall in the neonatal period. The blood flow in the abdominal wall muscular layer is maintained by the superior and inferior epigastric arteries, and the perforating branches of those arteries through the rectus abdominis muscle are the feeding vessels of the abdominal wall skin [16]. The anterior CST requires extensive dissection between the skin and anterior sheath of the rectus abdominis muscle. In our case, we kept such dissection to a minimum to preserve the perforating branches. We also performed surgery while taking care to preserve additional blood flow from the superficial epigastric arteries.

In a further attempt to avoid impairment of the blood flow due to a sudden increase in intra-abdominal pressure, the pressure was managed by controlling the abdominal wall tension with muscle relaxants and monitoring the intravesical pressure. We tried to maintain the pressure at < 12 mmHg, which is the upper limit of normal, and ensured that it did not exceed 20 mmHg, which is the diagnostic criterion for abdominal compartment syndrome reported in the literature [4]. An increase in intravesical pressure was observed in the early postoperative period, so muscle relaxant dosages were increased to relieve the tension in the abdominal wall, thereby avoiding the occurrence of skin necrosis due to an impaired blood flow.

## Conclusions

A giant omphalocele with primary skin closure was repaired using the anterior CST. The patient had a history of relaxing incisions and was considered to be at high risk for postoperative skin ischemia. By preserving the feeding vessels of the abdominal wall skin at the time of surgery and controlling the intra-abdominal pressure by measuring the intravesical pressure in the perioperative period, an impairment of the blood flow in the abdominal wall skin was prevented. In cases of giant omphalocele, the CST is considered to be effective for repairing large abdominal wall defects when primary closure is not possible.

## Data Availability

The data that support the findings of this study are available from the corresponding author upon reasonable request.

## Abbreviation

CST: Component separation technique

## References

[1] Bauman B, Stephens D, Gershone H, Bongiorno C, Osterholm E, Acton R (2016). Management of giant omphaloceles: a systematic review of methods of staged surgical vs. nonoperative delayed closure. J Pediatr Surg.

[2] Balla A, Alarcón I, Morales-Conde S (2020). Minimally invasive component separation technique for large ventral hernia: which is the best choice? A systematic literature review. Surg Endosc.

[3] Heller L, McNichols CH, Ramirez OM (2012). Component separations. Semin Plast Surg.

[4] Kirkpatrick AW, Roberts DJ, De Waele J, Jaeschke R, Malbrain ML, De Keulenaer B (2013). Intra-abdominal hypertension and the abdominal compartment syndrome: updated consensus definitions and clinical practice guidelines from the World Society of the Abdominal Compartment Syndrome. Intensive Care Med.

[5] Gottlieb M, Davenport DV, Adams S, Chien N (2019). Current approach to the evaluation and management of abdominal compartment syndrome in pediatric patients. Pediatr Emerg Care.

[6] Ramirez OM, Ruas E, Dellon AL (1990). “Components separation” method for closure of abdominal-wall defects: an anatomic and clinical study. Plast Reconstr Surg.

[7] Jernigan TW, Fabian TC, Croce MA, Moore N, Pritchard FE, Minard G (2003). Staged management of giant abdominal wall defects: acute and long-term results. Ann Surg.

[8] Poulakidas S, Kowal-Vern A (2009). Component separation technique for abdominal wall reconstruction in burn patients with decompressive laparotomies. J Trauma.

[9] Breuing K, Butler CE, Ferzoco S, Franz M, Hultman CS, Kilbridge JF (2010). Incisional ventral hernias: review of the literature and recommendations regarding the grading and technique of repair. Surgery.

[10] Wijnen RM, van Eijck F, van der Staak FH, Bleichrodt RP (2005). Secondary closure of a giant omphalocele by translation of the muscular layers: a new method. Pediatr Surg Int.

[11] van Eijck FC, de Blaauw I, Bleichrodt RP, Rieu PN, van der Staak FH, Wijnen MH (2008). Closure of giant omphaloceles by the abdominal wall component separation technique in infants. J Pediatr Surg.

[12] Vargo JD, Larsen MT, Pearson GD (2016). Component separation technique for repair of massive abdominal wall defects at a pediatric hospital. Ann Plast Surg.

[13] Kondra K, Jimenez C, Stanton E, Chen K, Shin CE, Hammoudeh JA (2022). Meeting in the middle: pediatric abdominal wall reconstruction for omphalocele. Pediatr Surg Int.

[14] van Eijck FC, van Vlimmeren LA, Wijnen RMH, Klein W, Kruijen I, Pillen S (2013). Functional, motor developmental, and long-term outcome after the component separation technique in children with giant omphalocele: a case control study. J Pediatr Surg.

[15] de Vries Reilingh TS, Bodegom ME, van Goor H, Hartman EH, van der Wilt GJ, Bleichrodt RP (2007). Autologous tissue repair of large abdominal wall defects. Br J Surg.

[16] Hellinger A, Roth I, Biber FC, Frenken M, Witzleb S, Lammer BJ (2016). Surgical anatomy of the abdominal wall. Chirurg.

